# Hepatocellular Brg1 promotes CCl4-induced liver inflammation, ECM accumulation and fibrosis in mice

**DOI:** 10.1371/journal.pone.0294257

**Published:** 2023-11-30

**Authors:** Baocai Wang, Benedikt Kaufmann, Carolin Mogler, Suyang Zhong, Yuhan Yin, Zhangjun Cheng, Roland M. Schmid, Helmut Friess, Norbert Hüser, Guido von Figura, Daniel Hartmann

**Affiliations:** 1 Department of Surgery, TUM School of Medicine, Klinikum Rechts der Isar, Technical University of Munich, Munich, Germany; 2 Department of General Surgery, The Affiliated Zhongda Hospital, School of Medicine, Southeast University, Nanjing, China; 3 Institute of Pathology, TUM School of Medicine, Klinikum Rechts der Isar, Technical University of Munich, Munich, Germany; 4 Department of Medicine II, TUM School of Medicine, Klinikum Rechts der Isar, Technical University of Munich, Munich, Germany; University of Hawai’i at Manoa, UNITED STATES

## Abstract

**Introduction:**

Hepatic fibrosis is a progressive pathological process involving the exhaustion of hepatocellular regenerative capacity and ultimately leading to the development of cirrhosis and even hepatocellular carcinoma. Brg1, the core subunit of the SWI/SNF chromatin-remodeling complex, was recently identified as important for liver regeneration. This study investigates the role of Brg1 in hepatic fibrosis development.

**Methods:**

Hepatocyte-specific Brg1 knockout mice were generated and injected with carbon tetrachloride (CCl_4_) for 4, 6, 8, and 12 weeks to induce liver fibrosis. Afterwards, liver fibrosis and liver damage were assessed.

**Results:**

Brg1 expression was significantly increased in the fibrotic liver tissue of wild-type mice, as compared to that of untreated wild-type mice. The livers of the Brg1 knockout animals showed reduced liver inflammation, extracellular matrix accumulation, and liver fibrosis. TNF-α and NF-κB-mediated inflammatory response was reduced in Brg1 knockout animals.

**Conclusion:**

Brg1 promotes the progression of liver fibrosis in mice and may therefore be used as a potential therapeutic target for treating patients with liver fibrosis due to chronic injury.

## 1. Introduction

Hepatic fibrosis is a stage of liver remodeling driven by various mechanisms of persistent liver injury and can be thought of as an excessive healing response to a wound. It is caused by hepatocyte necrosis, inflammation, and pathogenic extracellular collagen deposition [1]. Chronic liver injuries potentially cause progress from the stage of fibrosis, a reversible wound-healing process, to irreversible cirrhosis, hepatocellular carcinoma (HCC), and even ultimately liver failure [2]. Prolonged exposure to toxic substances can cause hepatocellular damage and apoptosis. Damaged hepatocytes release reactive oxygen species (ROS) and fibrotic mediators, such as nuclear factor kappa-B (NF-kB), tumor necrosis factor alpha (TNF-α), and transforming growth factor beta 1 (TGF-β1), which recruit inflammatory cells and leukocytes [3] and can promote the fibrotic response via different routes. NF-κB enhances liver fibrosis by promoting the survival of hepatic stellate cells (HSCs) [4]; TNF-α has a regulatory role in extracellular matrix remodeling and liver fibrosis [5]; and TGF-β1, which activates HSC, is the most potent known fibrogenic agonist [6]. Continuous release of these mediators sustains chronic hepatic inflammation that further activates profibrotic cells and subsequently promotes liver fibrosis [7]. Recent studies have shown epigenetic mechanisms that modulate different aspects of liver fibrogenesis. However, the exact role of the SWI/SNF family member Brg1 in this process remains unclear.

A previous study from our group demonstrated that Brg1, as the core ATPase of the SWI/SNF family, is overexpressed in patients with HCC and positively promotes proliferation and Brg1-regulated cell cycle pathway in liver regeneration [8]. Brg1 has also been reported to be involved in cardiac, renal, and liver fibrosis [9–12]. In the liver, Brg1 plays a key role not only during fibrosis but also in steatosis, a risk factor for developing liver fibrosis. Recent research has shown that hepatocyte-specific deletion of Brg1 suffices to alleviate steatosis in mice. Specifically, Brg1 functions as a coactivator for SREBP1c by laying down the optimal chromatin structure for SREBP1c target genes [13]. In the liver, hepatocytes cultured with free fatty acids were shown to overexpress Brg1 and Brm, thus stabilizing NF-κB, which is required for the development of steatosis, inflammation, and fibrosis in mice fed a methionine- and choline-deficient diet. Lentivirus-mediated knockdown of Brg1 attenuates steatosis in mice by downregulating the hepatic output of proinflammatory mediators via interfering with the NF-κB pathway [14]. Regarding liver fibrosis, endothelial Brg1 expression attenuates fibrogenesis by regulating ROS production [15]. Potentially also relevant for liver fibrosis, the profibrogenic TGF-β gene responses in human epithelial cells are dependent on Brg1 function [16]. In addition, hepatocyte-specific Brg1 expression can activate HSCs through a TGF-β/Smad signal pathway [17]. However, the exact role of hepatocellular expression of Brg1 in liver fibrosis development remains unclear. To address this in the current study, mice with liver-specific loss of Brg1 were analyzed using a carbon tetrachloride (CCl_4_) fibrosis model. The current study shows that knockout of Brg1 in mice attenuates the development of liver fibrosis by promoting a proinflammatory response. These results suggest that Brg1 or its targets could serve as a potential therapeutic target for liver fibrosis.

## 2. Results

### 2.1. Brg1 expression increases after CCl4 injection

**Fig 1:**
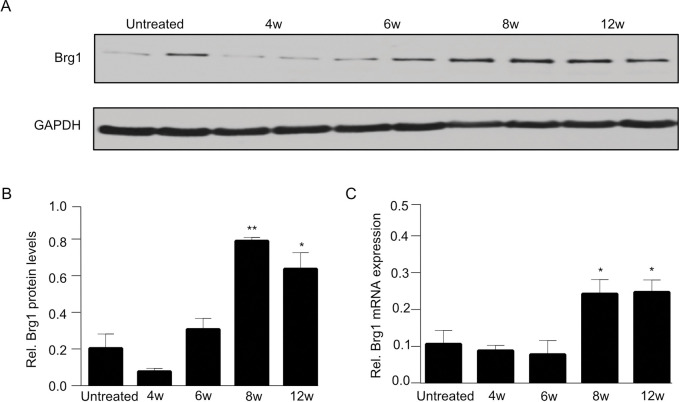
(**A**, **B**) Protein expression of Brg1 in the liver before and after CCl_4_ injection was analyzed by Western blot. Representative gels (**A**) and densitometric analyses (**B**) are depicted, n = 4. (**C**) mRNA expression of Brg1 after CCl_4_ injection, n = 4.

To induce liver fibrosis, mice were given intraperitoneal injections of the hepatotoxin CCl_4_. Hepatic Brg1 expression was assessed in mice, before and at different time points after the CCl_4_ injection. Weak Brg1 expression was observed in the healthy livers. At the early stage of liver fibrosis (4 and 6 weeks), no significant alterations existed in the Brg1 expression on mRNA or protein level (Fig 1A–1C). The protein and mRNA levels of Brg1 both started to gradually increase when liver fibrosis proceeded. Protein and mRNA levels of Brg1 showed the highest expression at 8 weeks after the CCl_4_ injections, (Fig 1A–1C). Brg1 protein expression increased approximately 4-fold after 8 weeks. These data indicate that Brg1 is upregulated in fibrotic livers, raising the hypothesis that Brg1 may play a functional role in the progression of liver fibrosis.

### 2.2. Brg1 knockout leads to a significant reduction of CCl_4_-induced liver fibrosis

**Fig 2:**
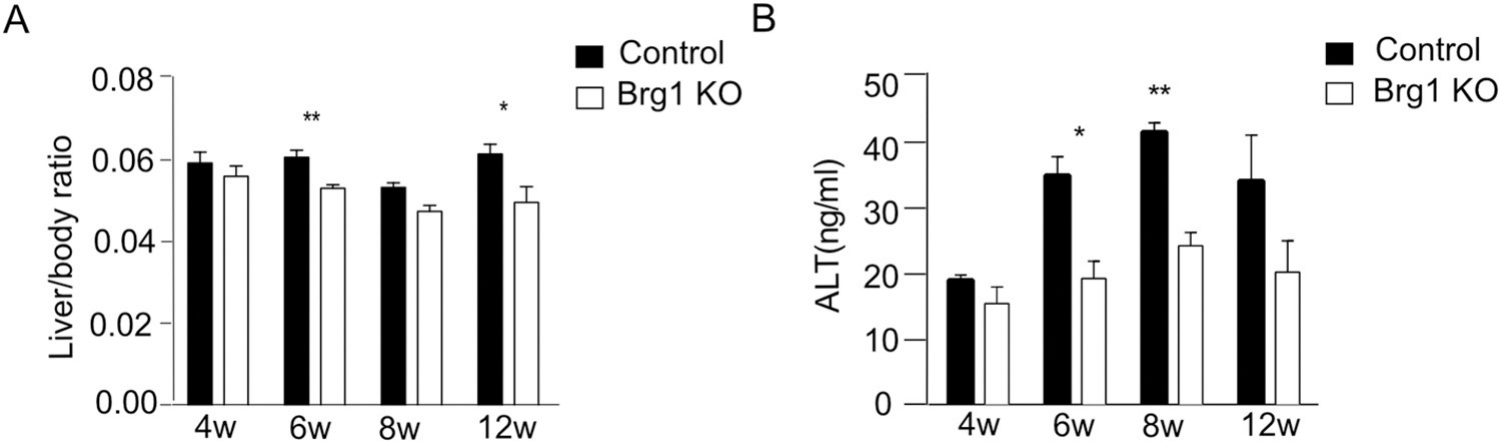
(**A**) Liver/body weight ratios were determined at the indicated time points after CCl_4_ injection, n = 6. (**B**) ALT levels were measured using serum samples of control and Brg1 KO mice after CCl_4_ injection, n = 3.

Next, the effects of Brg1 deletion on CCl_4_-induced liver injury and liver fibrosis were investigated. An increase in the liver/body ratio indicates liver injury. Over time (6 and 12 weeks after CCl_4_ injection), the liver/body weight ratio was significantly lower in *Alb-Cre; Brg1*^*f/f*^ mice (Brg1 KO group) compared to the control group (Fig 2A). Alanine- aminotransferase (ALT) in serum was examined to measure liver injury (Fig 2B). ALT levels were significantly higher in the serum of the control group than in the Brg1 KO group 6 and 8 weeks after CCl_4_ injection.

**Fig 3:**
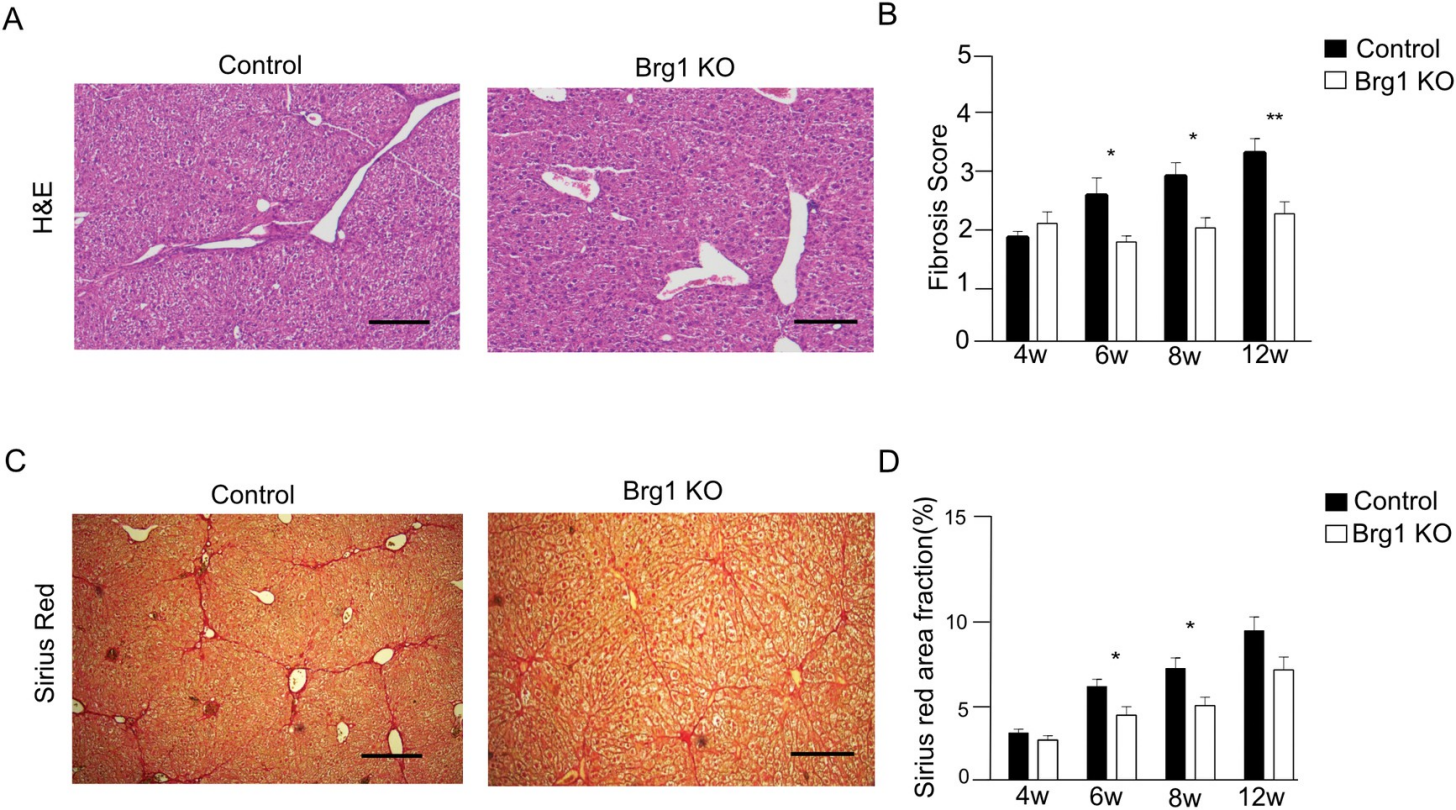
(**A**) Representative H&E staining of the liver of the control and Brg1 KO groups 8 weeks after the start of CCl_4_ treatment. (**B**) Quantification of fibrosis scores at the indicated time points, n = 6. (**C**) Representative Sirius Red staining of the liver of control and Brg1 KO group 8 weeks after CCl_4_ injection. (**D**) Quantification of Sirius Red area fraction at the indicated time points, n = 6, scale bar represents 200μm.

Sirius Red staining was used to assess the collagen fibrils in the liver tissues after CCl_4_ injection. Although the livers in both groups showed strong collagen deposition, the density of fibrous deposition in livers from the Brg1 KO group was significantly lower than that of the control group (Fig 3C and 3D).

**Fig 4:**
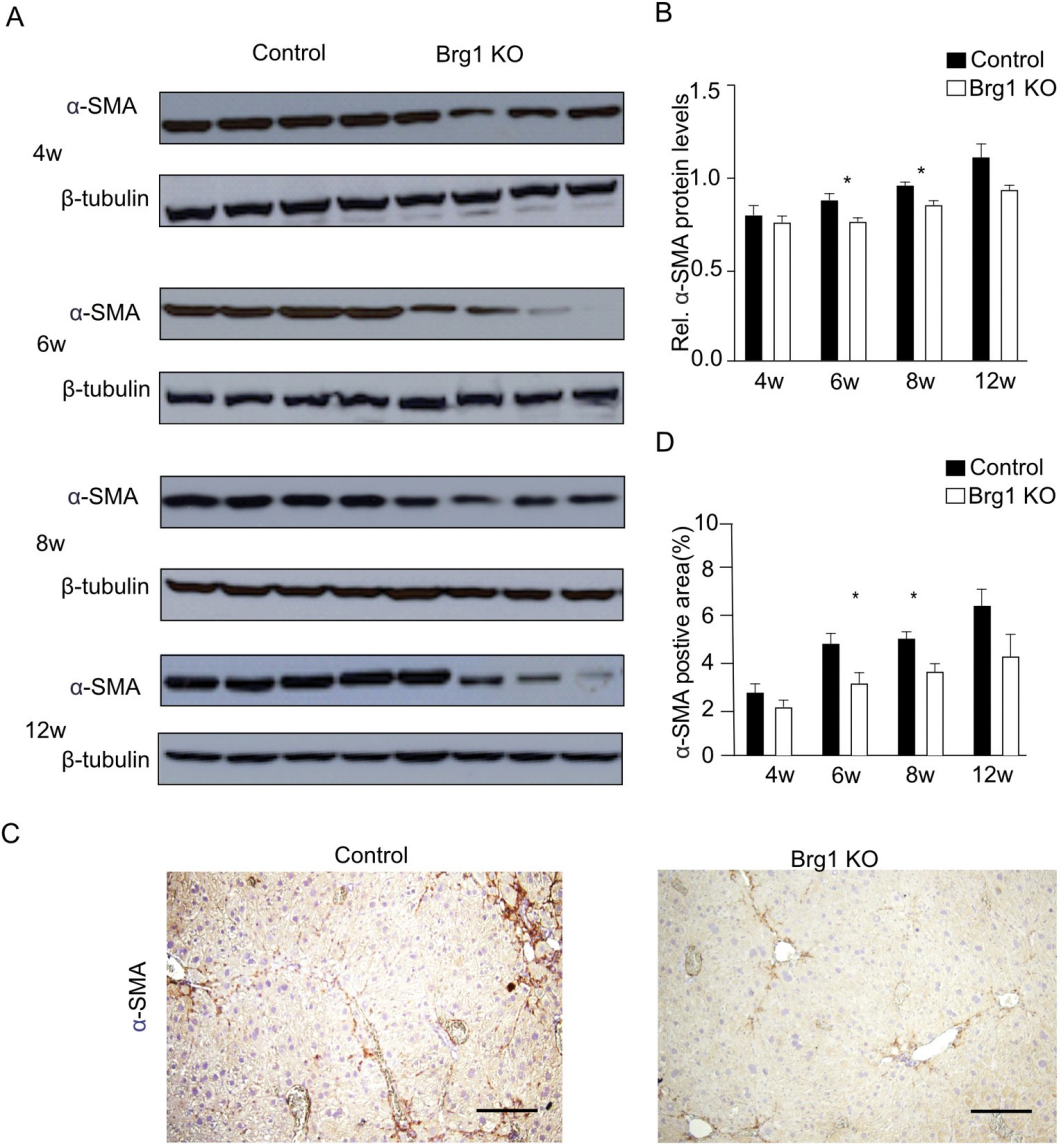
(**A**) Protein expression of α-SMA in the liver after CCl_4_ injection were analyzed by Western blot. Representative gels (**A**) and densitometric analyses (**B**) are depicted, n = 4. (**C**) Representative immunohistochemical images for α-SMA at 8 weeks after CCl_4_ injection in mice. (**D**) Quantification of α-SMA positive area at the indicated time points, n = 6, scale bar represents 200μm.

Hepatic fibrogenic response is associated with the transdifferentiation of HSCs into myofibroblasts. Alpha smooth muscle-actin (α-SMA) is a marker of CCl_4_-induced hepatic stellate cell activation. We used immunohistochemistry and western blotting to analyze the expression of α-SMA in control and Brg1 KO mice. Its relative expression in the Brg1 KO group was significantly lower than that in the control group 6 and 8 weeks after CCl_4_ injection (Fig 4A and 4B). The percentage of positive α-SMA area in the Brg1 KO group was also significantly lower than that of the control group 6 and 8 weeks after CCl_4_ injection (Fig 4C and 4D). Taken together, the α-SMA analysis revealed that the Brg1 KO mice had diminished α-SMA expression compared to the control group. This indicates that the Brg1 KO group had fewer activated hepatic stellate cells than the control group did.

### 2.3. Brg1 deletion suppresses TNF-α- and NF-κB-mediated inflammatory response in CCl_4_-induced fibrosis

The inflammatory mediator TNF-α and its downstream target NF-κB are expressed in different liver cell types and are involved in liver fibrosis [18]. We analyzed the protein expression of TNF-α and NF-κB to determine whether Brg1 hepatocyte deletion impacts the TNF-α/NF-κB pathway in CCl_4_-induced liver fibrosis.

**Fig 5:**
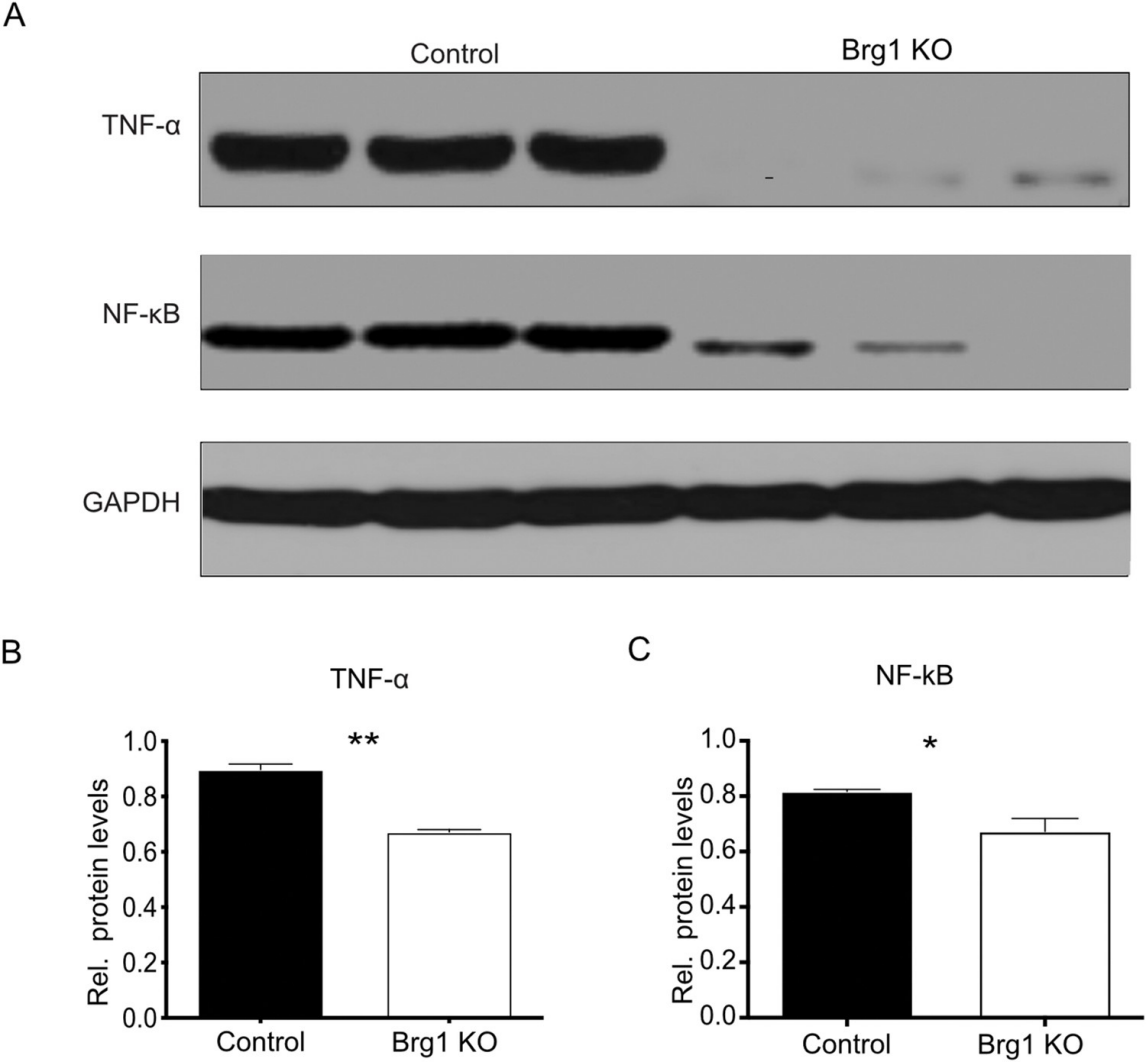
Protein expression of TNF-α and NF-κB in the liver after the CCl_4_ injections was analyzed by Western blot. Representative gels (**A**) and densitometric analyses (**B, C**) are depicted, n = 3.

TNF-α and NF-κB expression was lower in the Brg1 KO group than in the control group 8 weeks after the CCl_4_ injections (Fig 5).

**Fig 6:**
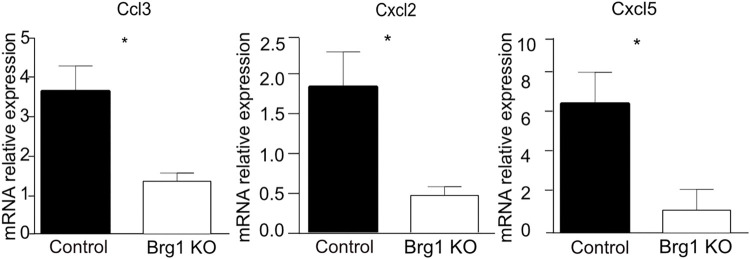
mRNA expression of Ccl3, Cxcl2, and Cxcl5 in the liver after CCl_4_ injection was analyzed by qPCR, n = 3.

In addition, the expression of genes involved in recruiting and maintaining inflammatory cells, including Ccl3, Cxcl2, and Cxcl5, was significantly upregulated in the livers of the control mice, as compared to the Brg1 KO mice, 8 weeks after the CCl_4_ injections (Fig 6). These data suggest that Brg1 promotes a TNF-α- and NF-κB-mediated inflammatory response.

## 3. Discussion

Liver fibrosis is a serious threat to the world’s population and is mainly driven by chronic liver injuries, which can subsequently lead to cirrhosis and HCC [19]. Studies have shown that Brg1 plays a crucial role in the fibrogenesis process for different diseases, mainly acting as a profibrotic gene [19]. The liver endothelium-specific conditional Brg1 knockout leads to an attenuation of liver fibrosis in mice by regulating ROS production [15]. In addition, Brg1 activates HSCs through TGFβ/Smad signal pathways [17]. However, Brg1’s exact role during fibrogenesis in the liver remains unclear. In this study, we investigated the effects of Brg1 on liver fibrogenesis and showed that hepatocyte-specific Brg1 expression promotes liver inflammation and fibrosis by promoting a proinflammatory response.

This study reveals that Brg1 expression increases in liver tissue during the progression of fibrosis. Furthermore, for the first time, hepatocyte-specific Brg1 knockout mice models were used to analyze the role of Brg1 in liver fibrosis. The deletion of Brg1 in hepatocytes suppresses the profibrotic response of liver inflammation and attenuates the progression of liver fibrosis. These findings of an upregulating and profibrotic role of Brg1 in liver fibrosis are in line with previous studies [19].

The activation of HSCs is considered to be an important marker of hepatic fibrosis, which is characterized by the upregulation of α-SMA and collagen [20]. The results of this study demonstrate that α-SMA and collagen expression are positively correlated with Brg1 expression, indicating an activation of HSCs via the Brg1 pathway during fibrogenesis. Genes involved in liver inflammation were analyzed to further reveal the mechanisms by which Brg1 activates HSCs. Liver tissue injury and liver inflammation are the initiating factors for liver fibrogenesis [7, 18]. Cell damage due to liver fibrosis releases chemokines and cytokines that cause inflammatory cell invasion. Thereby, the NF-κb pathway plays a key role as a proinflammatory signaling pathway. The NF-κb pathway is activated by proinflammatory chemokines and cytokines, including TNF-α [21]. Hereby, the interplay between NF-κb and Brg1 plays an essential role [22–24]. Brg1 interacts with histone-modifying enzymes to modulate the recruitment of NF-κb to the target promotors [22]. The direct functional complex formation of Brg1 and NF-κb has also been shown [24]. TNF-α is a key proinflammatory cytokine and chemokine that triggers an inflammatory cascade and further induces apoptosis in damaged hepatocytes [25]. Besides activating the NF-κb pathway, TNF-α can also directly activate HSCs, underlining its key role in fibrogenesis [18]. Interestingly, a direct activation of TNF-α by Brg1 has been shown previously [26]. This study demonstrated that the expression of the chemokines CCL3, CXCL2, and CXCL5 is reduced in hepatocyte-specific Brg1 KO mice during fibrogenesis. Previous studies have shown that these chemokines promote liver inflammation and fibrosis in mice [27–29]. In addition, this study reveals, for the first time, a positive correlation between TNF-α/NF-κb and Brg1 expression during liver fibrogenesis. These findings are in line with previous studies that showed a direct [30] activation of TNF-α and NF-κb by Brg1. Here, we have reported that TNF-α/NF-κb expression was reduced in hepatocyte-specific Brg1 KO mice, as compared to in control mice. Besides the reported activation of HSC via TGF-β/Smad signal pathways [17], the findings of this study reveal that Brg1 is important for the TNF-α/NF-κb pathway, which activates HSCs.

In conclusion, this study’s findings demonstrate a key role of hepatocyte-specific Brg1 expression during fibrogenesis. Brg1 also promotes the development of liver fibrosis by modulating liver inflammation via the TNF-α/NF-kB pathway. The exact mechanisms by which Brg1 and its downstream targets promote liver fibrosis are not completely understood and thus require further analysis. However, the findings of this study highlight Brg1’s role in liver fibrosis and provide a potential new therapeutic target for treating patients with liver fibrosis due to chronic injury.

## 4. Materials and methods

### 4.1. Animals

All of the mice were housed in specified pathogen-free facilities (ZPF, Klinikum rechts der Isar, Munich, Germany). Mice with a homozygous deficiency of Brg1 in their hepatocytes were generated by an intercrossing Brg1^fl/fl^ and AlbCre single mutant mice with a mixed genetic background. Corresponding controls (Brg1^fl/fl^, Brg1^fl/-^) were provided. We injected CCl4 (Sigma-Aldrich, Oakville, ON) (0.5 μl/g body wt) diluted in corn oil at a 1:7 ratio into the peritoneum (IP) of adult male mice (the age-matched controls were 8–10 weeks old). We gave the mice injections twice a week over 4-, 6-, 8-, and 12-week periods. Three days after the final injection, we euthanized the mice by neck dissection under anesthesia with inhalation of isoflurane and bleeding out, removed and weighed their livers, and either froze pieces of the livers for biochemical or molecular analyses or fixed them in 4% buffered paraformaldehyde for histochemical or immunohistochemical analyses. All efforts were made to alleviate suffering and mice were monitored closely after CCl4 injection. The animal experiments were institutionally approved by the district government of Upper Bavaria (AZ 55.2.1.54-2532-125-2015).

### 4.2. Liver function test

Blood samples were collected in heparinized tubes and allowed to clot at 4°C. The serum level of alanine transaminase was measured using an ELISA kit according to the manufacturer’s instructions (USCN Life, USA).

### 4.3. Western blotting

The liver samples were lysed in RIPA buffer (Cell Signaling Technology) supplemented with protease and phosphatase inhibitors. Protein concentrations were determined by using the Pierce^TM^ BCA Protein Assay Kit (Thermo Scientific). Lysates were separated by SDS-PAGE and transferred to Whatman Protran BA85 membranes (GE Healthcare). The membranes were incubated with the following primary antibodies overnight: Brg1 (Santa Cruz Biotechnology), α-SMA (Abcam), TNF-α (CST), NF-kB (CST), and GAPDH (Santa Cruz Biotechnology). Then, they were incubated with goat-anti-rabbit-HRP or goat-anti-mouse-HRP secondary antibodies (Promega) for 1 h. The antibody binding was visualized using the Pierce™ ECL western blotting detection system (GE Healthcare). Densitometric analysis was performed using the ImageJ software (https://imagej.nih.gov/ij/)).

### 4.4. Quantitative reverse transcriptase polymerase chain reaction

RNA was prepared using the RNeasy Mini Kit (Qiagen N.V.). First-strand cDNA was synthesized from 1 mg of total RNA using the QuantiTect Reverse Transcription Kit (Qiagen N.V.). All of the primers showed 90–100% efficiency and displayed a single melting curve. Quantitative RT-PCR (reverse transcription–polymerase chain reaction) analyses were performed using SYBR Green (Roche Diagnostics). The expression levels of specific genes were normalized to those of the housekeeping gene GADPH and were depicted as the fold difference relative to liver samples of untreated mice. The accumulation of PCR amplicons was quantified on a LightCycler 480 Real-Time PCR System (Roche Diagnostics).

### 4.5. Histology and immunohistochemistry

The liver samples were fixed overnight in 4% paraformaldehyde, dehydrated in a graded alcohol series, and embedded in paraffin. Hematoxylin and eosin (H&E), Sirius Red, and immunohistochemistry stainings were performed on 3μm archived liver sections. The primary antibodies used are was α-SMA (Abcam). The sections were stained with diaminobenzidin (DAB, Liquid DAB+ Substrate Chromogen System, DAKO). For each animal, five random high-power fields were counted, and the fractions of stained Sirius Red staining and SMA-positive areas were quantified using the Image J software.

### 4.6. Statistical analysis

Statistical analysis was performed using the GraphPad Prism software (5.0a; GraphPad Software, Inc., San Diego, CA). Variation was always indicated using standard errors, presented as mean ± SEM. Continuous data were tested for normality and analyzed with unpaired Student’s *t*-tests, Mann-Whitney *U* tests, or one-way ANOVAs, as appropriate. Statistical significance is displayed as *p* < 0.05 (*) or *p* < 0.01 (**), unless specified otherwise. In all of the experiments, no mice were excluded from the analysis after each experiment was initiated. Image analysis for the quantification was blinded.

## 5. Conclusions

In summary, this study demonstrates that Brg1 modulates liver fibrosis by promoting liver inflammation. It also shows that HSC activation and inflammation response during CCl_4_-induced liver fibrosis are associated with Brg1, which mediates the TNF-α/NF-kB pathway. These results highlight a new aspect of Brg1 in the pathogenesis of liver fibrosis. Thus, Brg1 inhibition appears to be a promising strategy for preventing hepatic fibrosis in patients with chronic liver diseases.

## Supporting information

S1 Raw images (PDF)
