# Supplementary material for: Hepatocellular Brg1 promotes CCl4-induced liver inflammation, ECM accumulation and fibrosis in mice
Source: PLoS One. 2023 Nov 30;18(11):e0294257. doi: 10.1371/journal.pone.0294257 (PMC10688683; doi:10.1371/journal.pone.0294257)

Figure 1A

Full length gels of all Western blots shown in the figures.

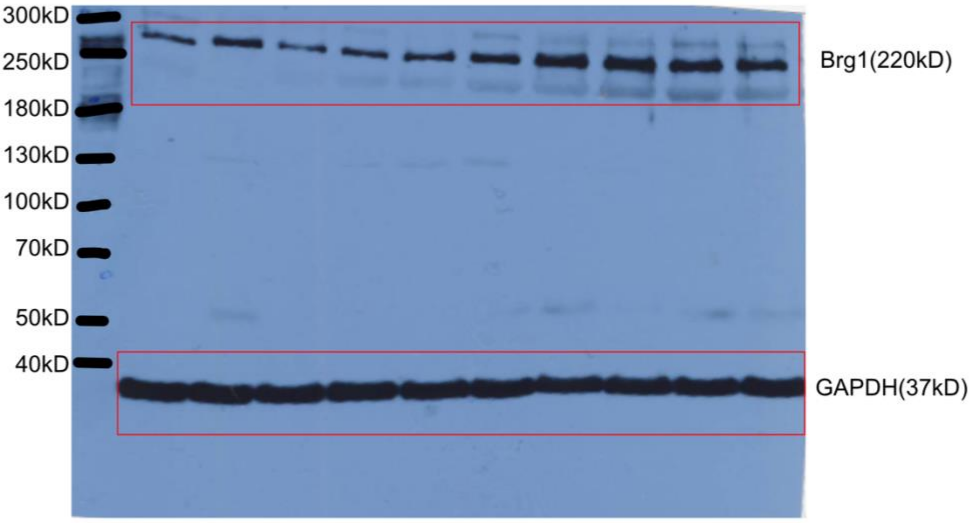

Figure 4A

Full length gels of all Western blots shown in the figures.

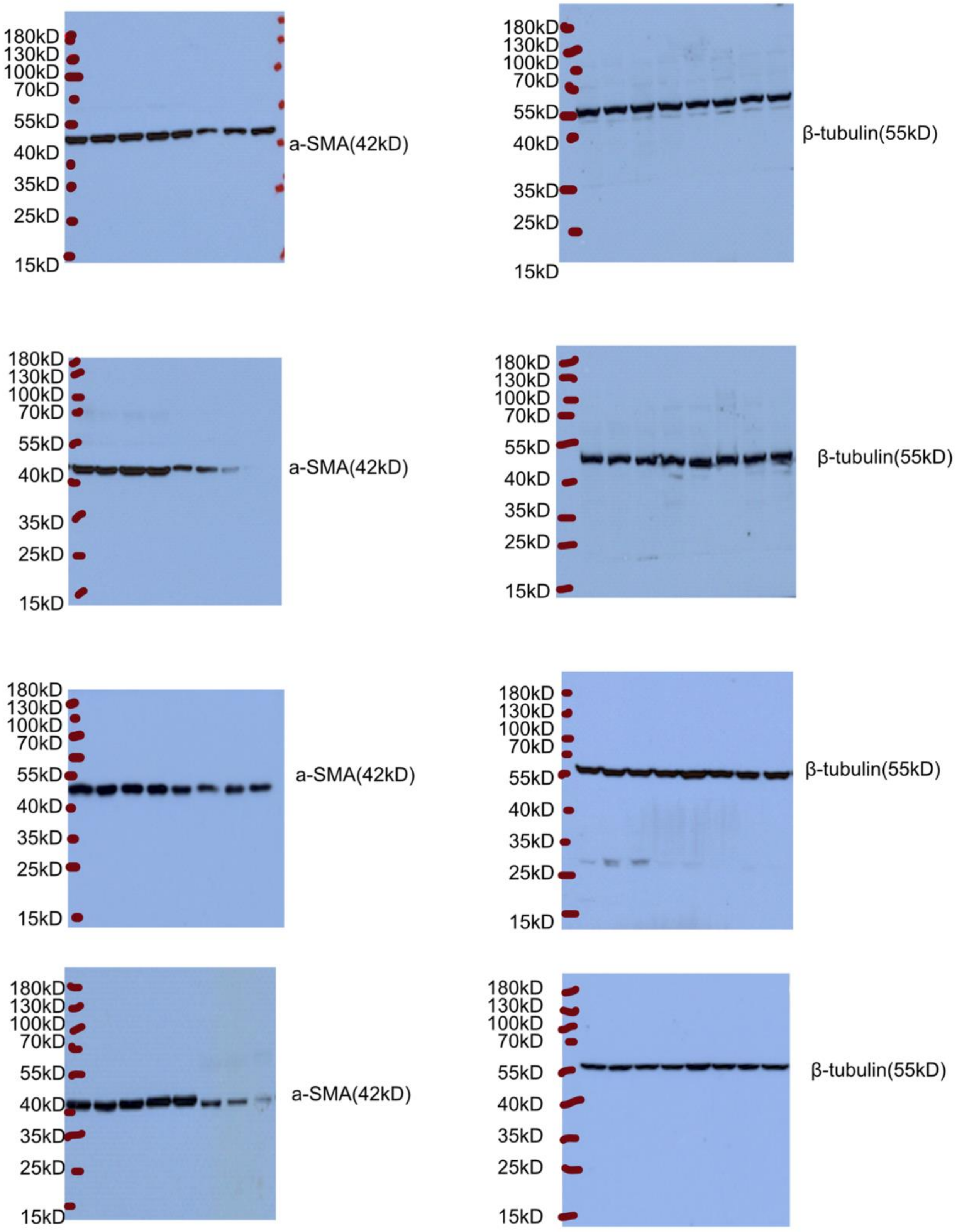

Figure 5A

Full length gels of all Western blots shown in the figures.

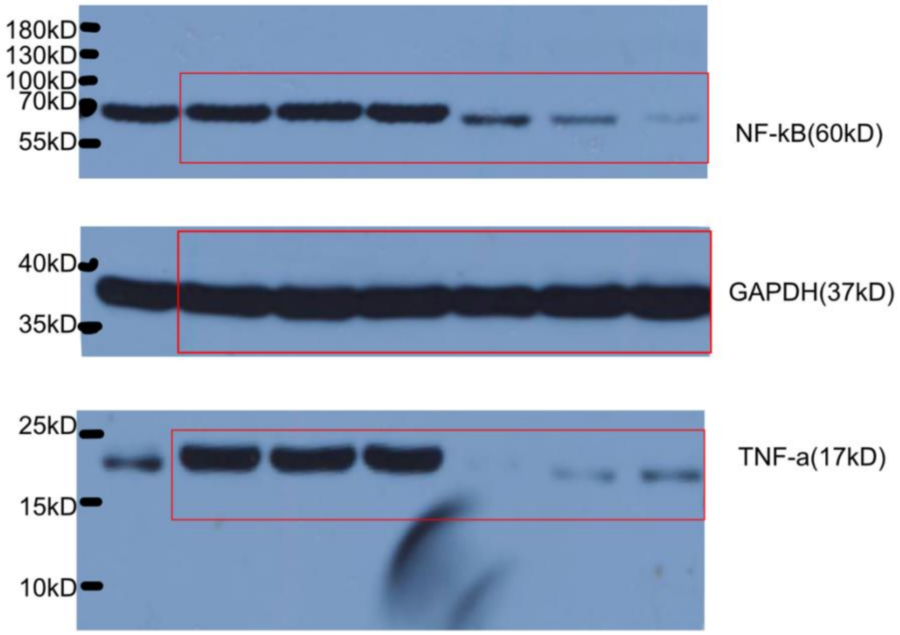

Supplement: S1 Raw images — (PDF) [file pone.0294257.s001.pdf]
